# The potential economic value of influenza vaccination for healthcare workers in The Netherlands

**DOI:** 10.1111/irv.12558

**Published:** 2018-05-24

**Authors:** Marjan J. Meijboom, Josien Riphagen‐Dalhuisen, Eelko Hak

**Affiliations:** ^1^ Unit of PharmacoTherapy, Epidemiology & Economics Groningen Research Institute of Pharmacy University of Groningen Groningen The Netherlands; ^2^ Department of Epidemiology University Medical Center Groningen Groningen The Netherlands

**Keywords:** economic value, healthcare workers, hospital, implementation, influenza, patients, vaccination

## Abstract

**Background:**

Despite the clinical evidence, influenza vaccination coverage of healthcare workers remains low. To assess the health economic value of implementing an influenza immunization program among healthcare workers (HCW) in University Medical Centers (UMCs) in the Netherlands, a cost‐benefit model was developed using a societal perspective.

**Methods/Patients:**

The model was based on a trial performed among all UMCs in the Netherlands that included both hospital staff and patients admitted to the pediatrics and internal medicine departments. The model structure and parameters estimates were based on the trial and complemented with literature research, and the impact of uncertainty explored with sensitivity analyses.

**Results:**

In a base‐case scenario without vaccine coverage, influenza‐related annual costs were estimated at € 410 815 for an average UMC with 8000 HCWs and an average occupancy during the influenza period of 6000 hospitalized patients. Of these costs, 82% attributed to the HCWs and 18% were patient‐related. With a vaccination coverage of 15.47%, the societal program’s savings were € 2861 which corresponds to a saving of € 270.53 per extended hospitalization. Univariate sensitivity analyses show that the results are most sensitive to changes in the model parameters vaccine effectiveness in reducing influenza‐like illness (ILI) and the vaccination‐related costs.

**Conclusion:**

In addition to the decreased burden of patient morbidity among hospitalized patients, the effects of the hospital immunization program slightly outweigh the economic investments. These outcomes may support healthcare policymakers’ recommendations about the influenza vaccination program for healthcare workers.

## 1 INTRODUCTION

Despite the available clinical evidence,^1^, ^2^, ^3^, ^4^, ^5^, ^6^, ^7^, ^8^ and the recognition that influenza vaccination has both a direct and an indirect medical effect, influenza vaccination coverage remains still very low among healthcare workers (HCWs).^2^, ^3^, ^4^, ^6^, ^8^, ^9^, ^10^ It has been demonstrated that vaccination decreases influenza infection rates among healthy adults, reduces the probability of viral transmission in healthcare settings, and indirectly benefits vulnerable patients by reducing the probability of becoming infected. The World Health Organization (WHO) recommends vaccination of all healthcare workers worldwide to protect staff and prevent potential transmission to their patients, but the response to this recommendation differs significantly between countries, professional organizations, advisory committees, and employers.^11^, ^12^, ^13^ In the United States, vaccination coverage among healthcare workers increased from 63.5% during the 2010‐2011 season to 77.3% during the 2014‐2015 season.^14^, ^15^, ^16^, ^17^ However, in Europe, healthcare workers are less compliant, with reported vaccine coverage as low as 30% or less.^18^, ^19^, ^20^ In general, the vaccination rate in the Netherlands and the United Kingdom is among the highest in Europe, although the coverage rate has been decreasing in the Netherlands since 2008 among the target population according to age (60 years and older) and/or certain medical conditions.^21^ Since 2008, the vaccination coverage decreased in previous years from 71.5% in 2008 to 52.8% in 2014.^19^, ^21^, ^22^ With the decreasing trend in the vaccine coverage rate of patients, it might become more important focusing on the vaccination rate of healthcare workers.

In long‐term care settings, four clinical trials are performed and despite the differences between the trials, they observed a decrease in patient morbidity or mortality after vaccine coverage increased.^2^, ^3^, ^4^, ^8^ For acute care settings, which treat patients during epidemics, the number of trials is limited. As these settings were not applicable to acute care settings, Riphagen et al performed a trial in acute care settings in the Netherlands.^7^, ^23^

Moreover, while influenza immunization is safe and relatively cheap, evidence on the economic benefits is not widely available for various healthcare settings, but this is an important aspect for hospital managers and policymakers to support such a program.^24^

To get a better understanding of the health economic benefits of a vaccination program for healthcare workers, we performed a modeling study using as much input as possible from a clinical trial and complemented information with additional data not provided by the trial.^7^, ^23^ The basis for the modeling study is a clustered randomized controlled trial, performed in the University Medical Centers in the Netherlands during the 2009‐2010 and 2010‐2011 influenza seasons.^7^ This study reports the health economic benefits of a vaccination program for healthcare workers, for an academic hospital with an occupancy of 6000 hospitalized patients during the influenza period and 8000 HCWs involved.

## 2 METHODS

### 2.1 Trial design and participants

The trial study design has been reported earlier.^7^ The study assessed the effects of a multifaceted influenza vaccination program in University Medical Centers (UMCs) in the Netherlands during two influenza seasons (2009‐2010 and 2010‐2011). In total, the hospital staff of three intervention UMCs (n = 27 900 in 2009), three control UMCs (n = 22 451), and two external non‐randomized intervention UMCs (n = 16 893) participated in the trial. In total, all 3367 patients admitted to the pediatrics and internal medicine department during both influenza epidemics participated in the trial.

### 2.2 Outcome measures

#### 2.2.1 Healthcare workers

The primary outcome measure of the trial was the influenza vaccine uptake among all HCWs at UMC level. Vaccine uptake was measured using the data of vaccinated persons, and this was divided by the total HCW population. Secondary outcome measures were absenteeism rates among HCWs during the month December of each study year as this was the month in which influenza peaked.^25^ The absenteeism rate was not extrapolated to the whole influenza season because of the rapid increase before, and the rapid decrease in the incidence after the month December, and to avoid substantial misclassification.^24^ These outcomes have been included in the present cost‐benefit study and completed with data from sources such as the average number of days of work absence, the number of GP visits following ILI, and the number of GP visit due to side effects following vaccination. The parameters are also expressed in monetary units as described in detail further below.

#### 2.2.2 Patients

As a secondary outcome, patient outcome data from two selected high‐risk departments (ie, Pediatrics and Internal Medicine) in the trial were collected retrospectively for all patients who were hospitalized 3 days or more to ensure nosocomial exposure during both study epidemic seasons. The outcomes were laboratory‐confirmed influenza and/or pneumonia, the length of hospital stay, use, and duration of intensive care and were collected by scrutinizing computerized discharge letters and laboratory outcome data from the microbiology laboratories by two reviewers. Influenza was defined as laboratory‐confirmed influenza A (all subtypes) or influenza B during a hospital stay. Pneumonia was defined as any pneumonia which was clinically diagnosed during a hospital stay.

Also, following the high mortality in risk groups during influenza epidemics, influenza mortality would be a valuable outcome measure. However, it appeared to be impossible to collect these data in the UMCs because of the absence of a good registration system for death. Thus, because it was impossible monitoring any mortality following hospital‐acquired pneumonia in the participating centers, it was decided not to include this in the calculations, and, therefore, the estimated outcomes can be considered conservative.

To estimate the effects on the reduction in the incidence of healthcare‐associated influenza and/or pneumonia for different vaccine coverage rates, a linear relationship between vaccine coverage rates of HCWs and the proportion of patients with outcomes was assumed according to the mathematical model by Van den Dool et al.^26^ In the estimates, an average of 23.7 per 100 additionally vaccinated HCWs in the intervention cluster as compared with the control cluster was assumed. The increase in coverage resulted in 2.7 per 100 fewer patients to develop influenza and/or pneumonia. Thus, if the coverage would be 70 per 100 HCWs (70% coverage), 7.8 per 100 fewer patients would develop influenza and/or pneumonia. This results in a 0.1139% decrease in the outcomes per 1% increase in vaccine coverage of HCWs. In other words, based on the trial data, it is calculated that per 1% increase in vaccine coverage of HCWs, 0.1139% less patients would develop influenza and/or pneumonia. In conclusion, patient‐related outcome measures included in the study are the number of patients with hospital‐acquired influenza and the costs for the influenza‐related treatment during the extended hospitalization.

### 2.3 Cost estimates

The cost estimates associated with the immunization program were based on Dutch guidelines for cost‐effectiveness research.^27^ The cost prices were indexed to the 2014 level.^28^ For healthcare workers, the direct medical costs and indirect non‐medical costs related to the research objective have been used and for patients the costs of the extended hospitalizations. For an overview of the model parameters used, see Table 1.

**Table 1:** Overview of the input parameters

Model parameters	Value	Reference
Number of employees in a hospital	8000	7
Total number of patients in a hospital	6000	7
Number of hospitalized patients and exposed to the same risk as patients in the departments where the clinical trial was performed)	600	Assumption
Vaccination coverage old (%)	0%	
Vaccination coverage new (%)	15.47%	7
Work absence due to ILI (%)	4.6%	7
GP visit following ILI	24%	29
Use of OTC with ILI	80%	29
GP visit due to side effects of the vaccination (%)	1%	29
Antibiotic use following GP visit (%)	20%	29
Decrease productivity ILI (in days)	4	1, 32, 33
Vaccine effectiveness in reducing ILI	20%	6
Probability of attracting influenza/pneumonia in hospital	11.34%	7
Cost		
Visit the GP	€30.78	27
Treatment of hospital‐acquired influenza/pneumonia	€1075	27, 29
Over‐the‐counter medicine	€7.02	29, 31
Cost of productivity loss per day	€225.82	7, 27
Antibiotic treatment	€7.62	29
Vaccination‐related costs	€15.00	24

### 2.4 Cost estimates for the healthcare workers

Costs associated with the immunization program, direct medical costs resulting from an influenza episode, and the effect vaccination has on the productivity are considered and the values used are substantiated further below.

#### 2.4.1 Costs associated with the immunization program and vaccine efficacy

The cost estimates of the influenza vaccination program were estimated at €15.00 per staff member and included the costs for the vaccine (approximately 5 euro), the communication, and implementation of the program.^24^ In the study by Hak et al,^24^ the potential cost savings were determined using plausible, but theoretical, effects in a UMC setting using the data from the University Medical Center Groningen. For the administration, a nurse gross salary (scale 9) per month was assumed with 5 minutes for vaccination of one staff member and another 5 minutes for correction of inefficiency (waiting time). The assumed costs currently assume a linear relationship between the number of persons vaccinated and the total cost for the vaccination campaign. Indirect costs due to productivity loss for the administration of the vaccine were assumed to be virtually absent because of the elasticity in working hours.

The vaccine efficacy for preventing ILI was assumed to be 20% because only vaccination status was available for HCWs and this was linked to the absence registry, but no laboratory‐confirmed influenza was measured.^6^

#### 2.4.2 Direct medical effect cost estimates

The direct medical effects of immunizing staff members against influenza are associated with seeking medical care for influenza. Direct medical costs associated with influenza were based on Dutch estimates from Postma et al^29^ and Hak et al^24^ in combination with data from a web‐based questionnaire carried out in 2009 and 2010 as part of the trial. The questionnaire was sent to all staff members of internal medicine and pediatrics as well as three other departments (two intensive care departments and neonatology), and the response rate was 31% in 2009 and 18% in 2010. The data from the various departments were pooled to increase statistical power on the outcome variables. The proportion of people seeking care at the general practice was estimated at 24% with an average of one GP consultation (€30.78). This corresponds with the outcomes of the research by Friesema et al that reports that around 20% of people with ILI visit the GP.^30^ Based on research by Postma et al*,* it was assumed that of all persons with ILI, 80% used over‐the‐counter (OTC) medications such as a nasal spray and paracetamol (€7.02) and 20% received an antibiotic at a price of €7.62 per course.^29^, ^31^ It was further assumed that vaccination in this healthy group would not lead to adverse events leading to hospital admission and that the vaccine caused side effects only in 10% of staff members resulting in associated GP consultations in 10% of them.^29^

#### 2.4.3 Working days lost due to influenza‐like illness

The productivity loss for the healthcare workers was calculated using the friction costs method. Studies reviewing the impact of influenza or influenza‐like illness on working days lost are very heterogeneous regarding the methodology used. Based on the available literature, 4 days working loss was used for influenza‐like illness.^32^, ^33^, ^34^, ^35^ Based on the work absence registration from the university hospitals, it was possible to calculate a gender and age‐weighted productivity costs per hour.^7^, ^27^ This resulted in an estimated average cost for 1 day of work loss of € 225.82.

### 2.5 Cost estimates for patients

The main patient‐related outcome was hospital‐acquired influenza and/or pneumonia and the costs related to the treatment and the extended hospitalization associated with the illness. For the cost‐benefit study, the indirect medical effect costs estimates were largely based on the costs associated with the occurrence of morbidity among patients and associated hospital care as observed in the trial. The average costs for this diagnosis were based on the estimated increased hospital stay of 1.7 days at the cost of €1013 for patients compared with the other hospitalized patients without nosocomial influenza and/or pneumonia.^36^

### 2.6 Cost‐benefit analysis

The decision tree was developed using Excel for Windows, version 2010. The deterministic probability analysis is based on trial data and on existing literature. In the analyses, both the societal perspective and the hospital perspective considered. Outcomes presented are incremental costs for the healthcare staff, the patients, total incremental costs, and the costs per extended patient hospitalization. The results are presented from a societal perspective, and the hospital perspective was also explored to consider aspects specifically relevant for the hospital as an employer.

Further, univariate deterministic sensitivity analyses were conducted to obtain the most influential model parameters on the outcome measure using plausible ranges. A multivariate probabilistic sensitivity analysis (PSA) with 10 000 iterations was performed to review the uncertainty of the model parameters simultaneously. The outcomes of the PSA are described in the results paragraph with the 95% confidence intervals (95% CI). The values used in the sensitivity analyses are presented in Table 1.

## 3 RESULTS

For an average UMC with 8000 staff members and 6000 patients, no vaccination of staff members was assumed in the base‐case scenario. In this scenario, 368 staff members were absent from work because of influenza‐like illness resulting in 1472 days of productivity loss, 88 staff members visited a general practitioner, 294 used OTC medications, and 18 an antibiotic treatment. Of the total costs of € 410 815, the direct medical care for staff members was estimated at € 4920 and € 332 407 for reduced productivity. Also, 70 patients developed influenza and/or pneumonia while hospitalized which resulted in a longer hospitalization. The cost associated with the extended hospitalization was estimated at € 73 488. Vaccinating 15.47% of the HCWs with a vaccine efficacy of 20% on ILI resulted in a reduction in work absenteeism for 11 HCWs and a reduction in the associated number of persons visiting a GP (3 persons), OTC use (9 persons), and persons using antibiotic treatment (1 person) but resulted in an increase in the number of persons visiting the GP due to the side effects of the vaccination (12 persons). The costs associated with the vaccination and the direct medical costs of the staff members increased with €18 793 and the costs for reduced productivity decreased with €10 285. In total, 11 patients were prevented from contracting influenza in the hospital, and this resulted in a reduction in the extended hospitalization period that could be valued at € 11 369. From a societal perspective, and thus including the effect of vaccination on both HCWs and patients leads to an incremental cost saving of € 2861. Dividing the total incremental cost saving by the number of prevented extended hospitalizations results in the total incremental saving per prevented extended hospitalization of a patient of € 270.53.

### 3.1 Hospital/employer’s perspective

From the hospital employer’s perspective, when only total influenza‐related costs for the healthcare staff are included, the total incremental investment for the vaccination program is € 8508 and the total cost per prevented extended hospitalization € 808.30. For an overview of the results from both perspectives, see Table 2.

**Table 2:** Results comparing 0% vaccination coverage with vaccination coverage of 15.47%

	Vaccination coverage (15.47%)	Vaccination coverage (0%)	Difference + 95% CI
Healthcare workers			
Employees (nr)	8000	8000	*0*
Nr employees vaccinated	1238	‐	*1238 (670.73; 1881.67)*
Nr persons absent from work	357	368	*−11 (−22.16; −4.05)*
Total nr days absent from work	1426	1472	*−46 (−99.77; −13.00)*
GP visit following ILI	85.59	88.32	*−2.73 (−5.64; −0.90)*
GP visits due to side effects of the vaccination	12.38	0.00	*12.38 (0.29; 46.87)*
Nr of persons with OTC use	285.29	294.40	*−9.11 (−17.78; −3.23)*
Nr of persons with antibiotic use	17.12	17.66	*−0.55 (−1.21; −0.17)*
Costs			
Vaccination	€18 564	€‐	*€18 564 (€8041.48; €33 807.36)*
GP visits following ILI	€2634	€2718	*€−84 (€−192.64; €−23.33)*
GP visits due to side effects of the vaccination	€381	€‐	*€381 (€8.47; €1 528.19)*
OTC use	€2003	€2067	*€−64 (€−139.98; €−19.09)*
Antibiotic use	€130	€135	*€−4 (€−10.10; €−1.07)*
Total direct medical costs	**€23 712**	**€4920**	***€18 793** (€8187.14; €34 177.12)*
Productivity loss	**€322 122**	**€332 407**	***€**−**10 285** (€−24 572.85; €−2567.70)*
Total costs healthcare workers	**€345 835**	**€337 327**	***€ 8508** (€‐5770.16; €23 627.73)*
Patients			
Nr of extended hospitalizations	58	68	*−11 (−20.37; −4.50)*
Costs of extended hospitalizations	€62 119	€73 488	*−€11 369 (€−24 709.83; €−4156.03)*
Total costs patients	**€62 119**	**€73 488**	***−€11 369** (€−24 709.83; € −4 156.03)*
Total costs (healthcare workers + patients)	**€407 954**	**€410 815**	*−**€2861** (€−21 828.36; €11 931.01)*

GP = general practitioner; ILI, Influenza‐like illness; OTC, over the counter.

### 3.2 Sensitivity analyses

**Figure 1:**
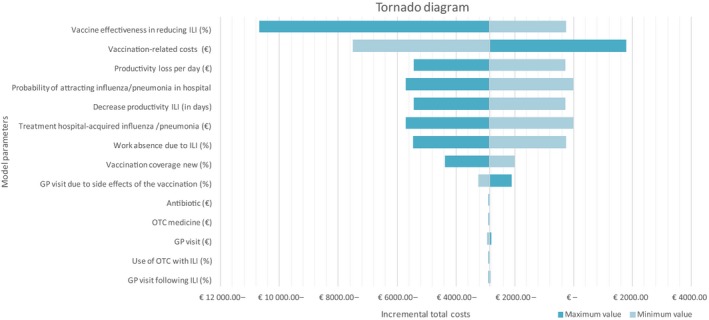
Tornado diagram

In the base case, when only the vaccination‐related costs are varied, the breakeven point is reached when the vaccination‐related costs are € 17.31 per vaccinated person. Above this cost, vaccinating healthcare workers is not cost saving from a societal perspective. From a hospital perspective, the vaccination‐related costs should not exceed € 8.13 to be cost saving. In case the work absence due to ILI is 25% lower (3 instead of 4 days), the vaccination program is breakeven (with a saving of € 289), but when the reduction in work absence due to ILI is lower than 3 days, the program is not cost saving anymore. Using the upper limit of the incremental vaccination coverage (23.7%) leads to a reduction in the disease burden of both healthcare workers and patients and a somewhat higher saving of € 4382. The lower limit of the incremental vaccination coverage (10.8%) results in a higher disease burden and, therefore, a lower saving of the vaccination program (€ 1997). The results of the univariate sensitivity analyses are presented in the tornado diagram and show that the model is most sensitive to changes in the parameters vaccine effectiveness in reducing ILI and the vaccination‐related costs. (Figure 1).

**Figure 2:**
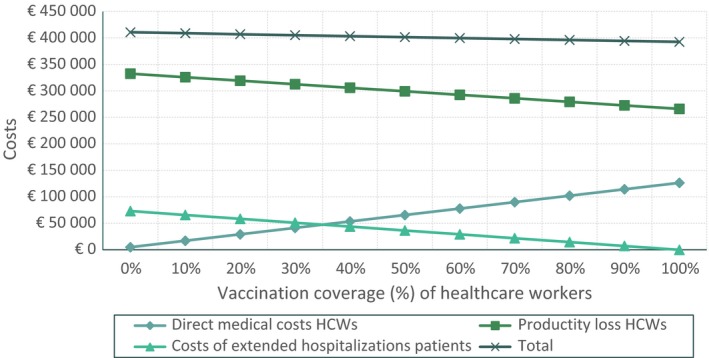
Costs related to vaccination coverage of HCW’s

The relationship between the vaccination coverage rate and the different identified cost components are explored and visualized in Figure 2 whereby incremental vaccination coverage rates between 0% and 100% show the development of the various costs. As the figure shows, in case of an incremental vaccination coverage of 40%, the incremental costs of productivity loss of healthcare staff equal the incremental costs of extended hospitalizations.

The multivariate probabilistic sensitivity analyses on the incremental total costs of both healthcare workers and patients show that the 95% confidence interval (95% CI) of the outcome incremental costs for both patients and healthcare workers correspond to average costs of—€2861 with 95% CI from—€21 828 and €11 931. For the outcomes of the PSA, see Table 2.

## 4 DISCUSSION

The aim of this study was to look at the health economic benefits of a vaccination program for healthcare workers, for an average academic hospital in the Netherlands. The study shows potential cost savings for society following the introduction of an influenza vaccination program among hospital staff. Main savings are the reduced productivity loss of the healthcare staff and in a minor part from reduced healthcare‐associated influenza infections for patients.

To value these findings, some aspects need to be considered. The input for the analysis was largely based on the established effects of the trial, and potential limitations and strengths of the trial have been discussed earlier but some relevant aspects for the cost‐benefit analyses are discussed here.

First, the absenteeism rate (4.6%) was estimated using the work absence registration from the hospitals participating in the trial. In the trial, a slight increase in absenteeism rates was reported in intervention as compared with control UMCs. It is likely a proxy for stricter regulations regarding working when staff has influenza and is not a result of the vaccination program. Therefore, it was decided to use the 4.6% for the situation where no vaccination was available. The vaccination coverage of 15.47% was lower than the median vaccination coverage reported by a European survey (25.7% in 2014‐15) but in line with a Dutch study by Gageldonk‐Lafeber et al.^7^, ^37^, ^38^ Mandatory vaccination policies as for example the US installed resulted in a vaccination coverage of hospital personnel of >90%.^39^ Taking the vaccination coverage of 90% into account in the model, the supposed saving would be €16 642 and 62 extended hospitalizations would be prevented.

Second, in the sensitivity analyses, the parameters vaccine efficacy and vaccination‐related costs were the parameters with the greatest influence on the results. The use of a vaccine effectiveness of 20% is considered appropriate as it is the best estimate available with the largest power, averaged over the largest number of years. It is not expected to lead to a significant over‐ or underestimation of the expected outcomes.^40^ The vaccination‐related costs included the cost of the vaccine, the program, and the time needed for vaccination and are measured in an earlier study. The Ministry of Health uses these estimates for the budget and reimbursement decisions.^24^, ^26^ Importantly, the current study is based on the results of conventional trivalent influenza vaccines, and in the future, studies are needed to assess the cost‐effectiveness and budget impact of other types of vaccines such as QIV, high dose, and universal vaccines.^41^

Third, the proportion of patients with healthcare‐associated nosocomial influenza and/or pneumonia was also an important cost driver. A considerable reduction was observed in the intervention vs control UMCs with an estimated 11.74% being infected during an epidemic. This figure agrees with the modeled 13% of nosocomial infection during an epidemic in a mathematical model developed by Van den Dool et al.^26^ It is also within a reasonable range of a study performed from 2006 to 2012 in a network of Canadian hospitals which reported 17.3% of healthcare‐associated influenza cases.^42^

Fourth, we did not include mortality rate following hospital‐acquired influenza because of the lack of a correct registration of the causes of mortality, but evidence suggests a higher mortality rate among patients with hospital‐acquired influenza.^2^, ^8^, ^43^, ^44^, ^45^, ^46^ For example, Salgado et al^45^ reported a median mortality rate of 16% among all patients and 33%‐60% in high‐risk groups such as transplant recipients and patients in the ICU. Taking the median mortality rate among all patients into account (16%) in the present study would mean that per hospital, 1‐2 patients of the 11 patients with hospital‐associated influenza would die which would have a significant impact on the study outcomes. The difficulties surrounding the mortality rate are confirmed by the systematic review performed by Ahmed et al.^47^, ^48^ They graded the available clinical evidence of influenza vaccination of healthcare workers on patients and healthcare workers and concluded that the quality of the evidence for the effect of HCW vaccination on mortality and influenza cases in patients was “moderate” and “low.” Thus, including the mortality rate is expected to be of importance, but additional research is necessary to substantiate the influenza‐associated mortality rate further. Consequently, the patient‐related results presented in this study are conservative estimates.

Finally, the trial included both an influenza pandemic and an epidemic. It is expected that the pandemic and the anxiety among healthcare workers have affected the trial results and, therefore, the robustness of the results presented here. However, this was the case in both the intervention and control group, and still, statistically significant differences were found between both groups. The influenza attack rate included in the calculations affects the outcomes of both patients and HCWs. In 2009, during the pandemic, the influenza attack rate was somewhat higher than generally, and during the 2010/2011 season, it was low. Because the average of the two seasons is used in the calculations, it is expected that a general epidemic was simulated.

## 5 CONCLUSION

The vaccination program is likely cost saving from a societal perspective. From the hospital perspective, it requires an investment by the hospital management, but the biggest return on investment is also for the hospital. However, these investments are not supported by financial incentives in the current system.

Studies are warranted that focus on the effect of vaccination programs in peripheral hospitals, on the costs for hospitals involved when preparing and organizing the influenza vaccination and on the effect of influenza vaccination on the mortality rate.
